# A Real-Time PCR Antibiogram for Drug-Resistant Sepsis

**DOI:** 10.1371/journal.pone.0028528

**Published:** 2011-12-02

**Authors:** John R. Waldeisen, Tim Wang, Debkishore Mitra, Luke P. Lee

**Affiliations:** Department of Bioengineering, University of California, Berkeley, California, United States of America; University of Alabama-Birmingham, United States of America

## Abstract

Current molecular diagnostic techniques for susceptibility testing of septicemia rely on genotyping for the presence of known resistance cassettes. This technique is intrinsically vulnerable due to the inability to detect newly emergent resistance genes. Traditional phenotypic susceptibility testing has always been a superior method to assay for resistance; however, relying on the multi-day growth period to determine which antimicrobial to administer jeopardizes patient survival. These factors have resulted in the widespread and deleterious use of broad-spectrum antimicrobials. The real-time PCR antibiogram, described herein, combines universal phenotypic susceptibility testing with the rapid diagnostic capabilities of PCR. We have developed a procedure that determines susceptibility by monitoring pathogenic load with the highly conserved 16S rRNA gene in blood samples exposed to different antimicrobial drugs. The optimized protocol removes heme and human background DNA from blood, which allows standard real-time PCR detection systems to be employed with high sensitivity (<100 CFU/mL). Three strains of E. coli, two of which were antimicrobial resistant, were spiked into whole blood and exposed to three different antibiotics. After real-time PCR-based determination of pathogenic load, a ΔC_t_<3.0 between untreated and treated samples was found to indicate antimicrobial resistance (*P*<0.01). Minimum inhibitory concentration was determined for susceptible bacteria and pan-bacterial detection was demonstrated with 3 Gram-negative and 2 Gram-positive bacteria. Species identification was performed via analysis of the hypervariable amplicons. In summary, we have developed a universal diagnostic phenotyping technique that assays for the susceptibility of drug-resistant septicemia with the speed of PCR. The real-time PCR antibiogram achieves detection, susceptibility testing, minimum inhibitory concentration determination, and identification in less than 24 hours.

## Introduction

Current molecular diagnostic technologies for septicemia have primarily focused on pathogen identification as a means to optimize antimicrobial therapy for patients. Such developments have included multiplexed PCR [1], [2], microarray platforms [3], [4], and chemiluminescent RNA probes [5] for the genetic detection of known bacterial and fungal organisms responsible for septicemia. Virulence and susceptibility are often deduced upon species identification as only a handful of known resistance genes can be screened with current commercial systems [2]. Compounding the issue of genotyping for resistance is the fact that the number of potential resistance genes scale with each pathogen, thus straining the limits of current diagnostic technology and economic feasibility. Despite this constraint, molecular diagnostic systems have demonstrated species identification in less than 24 hours, a drastic improvement in comparison to the gold standard culture-based susceptibility and Gram staining-based identification methods that yield results in 24 to 72 hours [3], [4], [6], [7]. However, even though literature agrees that molecular diagnostic detection rapidly decreases the time to sepsis diagnosis, much debate over the accuracy of pathogen identification and hence, the appropriateness of the method for prescribing antimicrobial therapy remains [1], [2], [8]–[12].

The anticipations of molecular diagnostic systems becoming the new gold standard for patient diagnosis have largely gone unmet and blood culture still remains as the *de facto* method to determine the course of patient treatment [2], [8]–[12]. In light of the focus on molecular diagnostics, efforts to develop rapid techniques to directly test pathogen susceptibility are lacking [13]. In fact, we believe direct susceptibility testing of such blood-borne pathogens may be of greater importance than species identification via genotyping as the selective pressures of broad-spectrum antibiotic use have contributed to the increased incidence and virulence of antimicrobial resistant septicemia [14]–[18]. Universal susceptibility testing by genotyping for resistance is inferior to traditional phenotypic methods, as the microorganisms must be screened for a multitude of resistance cassettes. This approach has intrinsic vulnerabilities as more complicated and genetically diverse mechanisms of antimicrobial resistance have remained elusive [18], [19]. Herein, we describe a method of direct susceptibility testing by monitoring phenotypic bacterial load via real-time PCR of spiked blood samples post-exposure to an array of antimicrobials. This method combines rapid molecular diagnostic detection with the traditional benefits of phenotypic testing to achieve universal susceptibility analysis, minimum inhibitory concentration determination, and pathogen identification in blood in less than 24 hours.

## Results and Discussion

### Experimental Overview

The real-time PCR antibiogram utilizes antimicrobial exposure, preanalytic removal of heme and human background DNA, and colony PCR to assess pathogen susceptibility [20], [21]. The optimized protocol is described below in the Materials and Methods Section. Briefly, 1 mL of spiked blood (∼100 CFU/mL) is added to 9 mL of growth medium and incubated for 9 hours in various antibiotic environments. The sample is fractionated to separate red blood cells that contain heme, a PCR inhibitor. The supernatant, which consists of bacteria and mammalian cells, is pelleted and decanted. The pellet is then resuspended in mammalian lysis buffer and treated with DNase. This technique removes human DNA found in white blood cells from the sample, thus enhancing the sensitivity of detection. This is an essential part of this protocol as excess background human DNA can saturate the PCR amplification curves when using intercalating fluorophores. The sample is spun-down and the unseen bacterial cell pellet is washed in reticulocyte saline (RS) buffer. Preparation concludes by adding 2 µL of the sample directly to the PCR plate as template. This bacterial isolation method takes approximately 2–3 hours of manual labor, but could be automated to decrease sample preparation time and multiplexed for high-throughput testing in a clinical diagnostic laboratory setting.

A target pathogen concentration of ∼100 CFU/mL was chosen to emulate levels found clinically in sepsis cases [22]–[24]. For bacteremia, greater than 50% of cases are low-grade (<10 CFU/mL) and ∼25% of cases are high-grade bacteremia (>100 CFU/mL), thus necessitating a detection sensitivity of at least 100 CFU/mL.

### The Real-time PCR Antibiogram

**Figure 1:**
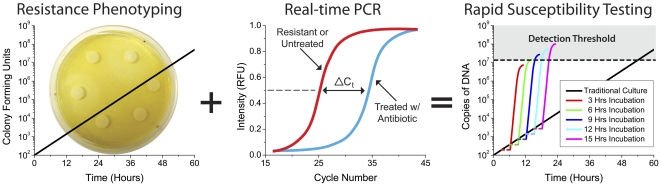
The real-time PCR antibiogram achieves rapid susceptibility testing of septicemia in less than 24 hours. Schematic that illustrates how phenotypic response can be monitored with the diagnostic speed of PCR to yield drastically decreased detection times. The real-time PCR antibiogram (middle) is determined by measuring the change in ΔC_t_ values for different antibiotics against a control. Parameters for the simulation (right) were optimized using experimental data and incubation times greater than 8+ hours allowed our detection system to assay cultures at ∼100 CFU/mL. The three-hour horizontal dashes before amplification depict manual sample preparation time.

Real-time colony PCR is performed using universal forward and reverse primers specific for the highly conserved region of the 16S ribosomal RNA gene sequence (Table 1) [25]. Real-time PCR enables the comparison of bacterial load after antibiotic exposure by monitoring the amount of 16S rDNA present. The Bio-Rad iQ5 real-time PCR detection system used in this investigation was determined to have a detection limit of approximately 2×10^4^ CFU/mL, thus necessitating an incubation time of at least 8 hours. Figure S1 depicts the serial dilutions and incubation time curves used to determine the colony PCR detection limit to optimize the procedure for sample preparation. The real-time PCR antibiogram method determines susceptibility in less than 24 hours, an improvement upon traditional phenotypic methods such as CO_2_ monitoring and agar dilution methods, which require 24 to 72 hours. The decreased time to detection using the real-time PCR antibiogram method is depicted in Figure 1 and illustrates how phenotypic response can be combined with the diagnostic speed of PCR to yield drastically decreased detection times of antibiotic resistance. The real-time PCR antibiogram (middle) is determined by measuring the change in ΔC_t_ values for different antibiotics against an untreated control. Variations in bacterial load post-exposure to an array of antibiotics are amplified by PCR, thus allowing pathogen detection and susceptibility to be obtained in much less time. Parameters for the simulation were optimized using experimental data and incubation times greater than 8+ hours allowed our detection system to assay cultures at ∼100 CFU/mL. The three-hour horizontal dashes before amplification depict manual sample preparation time.

**Table 1:** Universal primers for the 16S rRNA gene.

Primer	Sequence (5′ → 3′)
Forward	AGAGTTTGATCMTGGCTCAG
Reverse	CTGCTGCSYCCCGTAG

### Susceptibility and Minimum Inhibitory Concentration Testing

**Figure 2:**
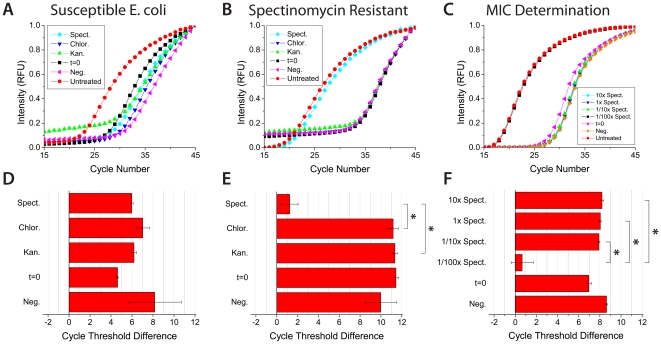
The real-time PCR antibiogram for aiding antimicrobial selection and administration. a-c) Real-time PCR amplification curves for measuring the susceptibility of susceptible, spectinomycin resistant, and the minimum inhibitory concentration of E. coli spiked in blood. Amplification curves were run in triplicate. d-f) The corresponding ΔC_t_ values for the real-time amplification curves. ΔC_t_ values >3.0 were assigned to indicate susceptibility, while ΔC_t_ values <3.0 designate resistance. *P<0.01 for multiple comparisons by the Holm *t* Test. t = 0 signifies initial bacterial levels with no incubation and negative curves denote sample preparation with no bacteria. Susceptible E. coli was used for minimum inhibitory concentration determination and 1x designates a spectinomycin concentration of 50 µg/mL.

The developed assay was performed on three strains of E. coli, two of which are resistant, for three types of antibiotics: kanamycin, spectinomycin, and chloramphenicol (Figure 2 and Figure S2). The real-time PCR antibiogram determines resistance by monitoring the relative difference in pathogenic bacterial load between treated and untreated blood samples. The cycle threshold difference (ΔC_t_) is defined as:
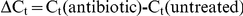



All experiments were run in triplicate, normalized, and the C_t_ value was defined as the cycle number in which the amplification curve crossed 0.5. ΔC_t_ values >3.0 were assigned to indicate susceptibility, while ΔC_t_ values <3.0 designate resistance. We observed no inhibition of PCR amplification from residual Heme or from any of the three antibiotics. Susceptible E. coli produced low bacterial loads when exposed to all three of the antibiotics and large ΔC_t_ values indicated susceptibility (Figure 2A and 2D). Spectinomycin resistant E. coli produced low bacterial loads to all the antibiotics except when incubated in the presence of spectinomycin, thus yielding a significantly lower ΔC_t_ value (Figure 2B and 2E). Similarly, kanamycin resistant E. coli produced the same result when incubation was performed with kanamycin (Figure S2). The real-time PCR antibiogram matrix summarizes the susceptibility results, as depicted in Table 2. Comparison of spectinomycin ΔC_t_ values between susceptible and spectinomycin resistant E. coli confirmed a significant statistical difference (Information S1 and Figure S3). Next, minimum inhibitory concentration was determined for blood samples spiked with E. coli susceptible to spectinomycin. The samples were incubated in four different concentrations of spectinomycin and there is a significant difference in the ΔC_t_ value at concentrations below the minimum inhibitory concentration (Figure 2C and 2F). Figure 2 illustrates how the real-time PCR antibiogram can determine the optimal patient-specific antimicrobial concentration for administration in addition to aiding antimicrobial selection.

**Table 2:** Real-time PCR antibiogram matrix for susceptibility testing.

	Kanamycin	Spectinomycin	Chloramphenicol
Susceptible E. coli	+6.2	+6.0	+7.0
Kanamycin Resistant	**−1.0**	+6.3	+5.7
Spectinomycin Resistant	+11.2	**+1.0**	+11.1

It is important to note that the cutoff value for ΔC_t_ is dependent upon factors intrinsic to the real-time PCR detection system employed, such as the cycle time, amplification efficiency of the polymerase, and range of the fluorescent detection window of the system. Thus we aim to provide a conceptualized approach to determining resistance that will need further clinical optimization. We anticipate that advanced stages of sepsis will have higher ΔC_t_ values needed to indicate resistance as pathogenic load generally correlates with the severity of sepsis (i.e. SIRS vs. sepsis vs. septic shock) and larger pathogenic loads will have greater deviation between patients. Cases in which there is partial antibiotic resistance, as with efflux pump upregulation and partial resistance to glycopeptide antibiotics conferred by the thickening of the bacterial cell wall, MIC determination would ascertain the antibiotic concentration or combination of antibiotics necessary for therapy.

### Pan-bacterial Detection and Identification

**Figure 3:**
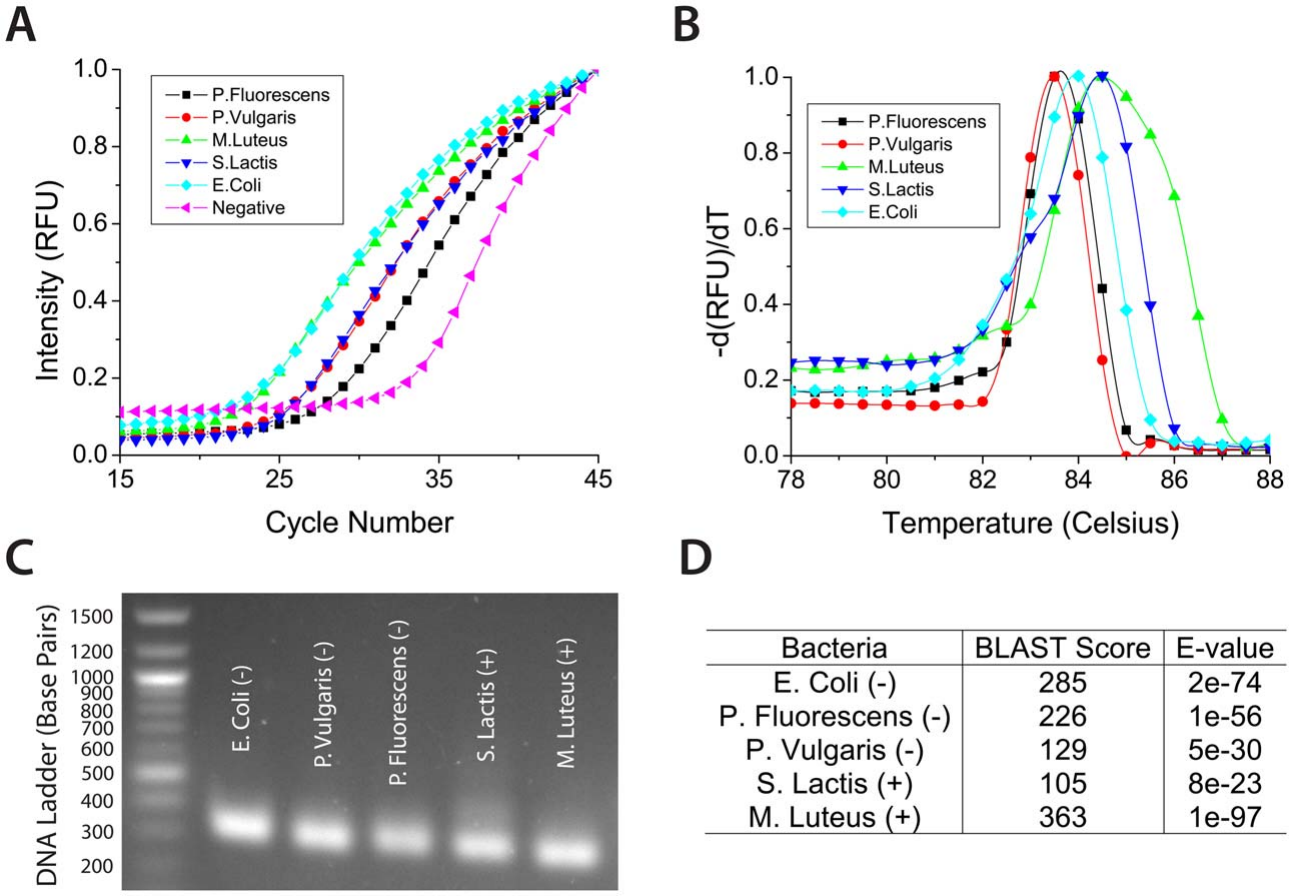
The real-time PCR antibiogram for pan-bacterial detection and identification. a) Pan-bacterial amplification illustrates universal binding of the primers and b) melt curve analysis provides evidence that consistent, species-specific amplicons were generated. c) Amplicons run on gel electrophoresis exhibit similar lengths of 200-300 base pairs and d) sequences submitted to the BLAST are readily identifiable.

Given the large population of microbes that can cause sepsis, pan-bacterial amplification was demonstrated with 3 Gram-negative and 2 Gram-positive bacteria (Figure 3A). Melt curve analysis illustrated how the primers produced consistent, species-specific amplicons (Figure 3B). The amplified 16S rDNA amplicons, which are approximately 200-300 base pairs in length, contain hypervariable regions that are capable of enabling species-specific, bacterial identification (Figure 3C). Sequenced amplicons were submitted to the BLAST database and the original species were readily identifiable (Figure 3D). Species identification could be equally obtained using multiplexed molecular probes found in current commercial diagnostic PCR systems.

### Conclusion

Recent innovations in susceptibility testing have largely failed to transcend the gold standard culture-based methods that have dominated clinical diagnostic laboratory protocols for the past 50+ years. Susceptibility testing of septicemia relies on either extended multi-day phenotypic methods that jeopardize patient survival or molecular genotyping techniques that are limited by the number of resistance cassettes the system can detect and that are unable to detect newly emergent resistance genes. These issues have resulted in the pervasive administration of broad-spectrum antimicrobials and the increased incidence and virulence of antimicrobial resistant septicemia.

By combining universal phenotypic susceptibility testing with the rapid diagnostic capabilities of PCR, we have developed a superior method of direct susceptibility testing. The procedure determines susceptibility by monitoring pathogenic load with the highly conserved 16S rRNA gene in blood samples exposed to different antimicrobial drugs. The optimized protocol removes heme and human background DNA from blood, which allows standard real-time PCR detection systems to be employed with high sensitivity (<100 CFU/mL). The real-time PCR antibiogram achieves detection, susceptibility testing, minimum inhibitory concentration determination, and identification in less than 24 hours. We anticipate the uptake of this technique to continue with subsequent clinical testing and comparison to the current standards of sepsis diagnosis and treatment.

## Materials and Methods

### Pre-analytic Removal of Heme and Human DNA Protocol

Defibrinated sheep blood (Hemostat Laboratories) was spiked with serial dilutions of bacteria and plated onto LB-Agar (Fisher Scientific) plates to determine initial bacteria concentrations in CFU/mL. Susceptible bacteria: Escherichia coli (ATCC No. 53338), Pseudomonas fluorescens (ATCC No. 13525), Proteus vulgaris (ATCC No. 33420), Micrococcus luteus (ATCC No. 49732), and Streptococcus lactis (ATCC No. 11454) (Fisher Scientific), and resistant E. coli (donated by J. Christopher Anderson Laboratory) were spiked into blood at concentrations ranging between 50–200 CFU/mL. 1 mL of spiked blood was added to 9 mL of LB Broth (Fisher Scientific, Miller) and grown in a shaker at 37°C in the presence of kanamycin, spectinomycin, or chloramphenicol (Sigma-Aldrich). The blood/LB mixture was then fractionated with a Beckman Coulter Allegra X-22R centrifuge for 10 min at 1500 rpm (524 g). This important step enables the removal of the PCR inhibiting Heme from the sample. 9.5 mL of supernatant, which consisted of bacteria, white blood cells, platelets, and serum, was collected and pelleted down for 10 min at 4500 rpm (4713 g). The supernatant (∼9 mL), which contained serum and platelets, was decanted and the remaining pellet was resuspended. 125 µL of RIPA buffer (Thermo Scientific) was added to lyse the mammalian cells, the solution was vortexed, and incubated at 25°C for 5 min. 20 µL of DNase 10x reaction buffer and 20 µL (1 U/µg) of DNase (Fisher Scientific) were added to remove human background DNA. The sample was vortexed and incubated at 37°C for 30 minutes. 20 µL of DNase stop solution was then added to inhibit the reaction. The bacterial cells were then pelleted from the lysate for 10 min at 4500 rpm (4713 g). It is important to note that no actual pellet was visible after centrifugation as the bacterial cell volume was inappreciable. The supernatant (∼600 µL) was decanted and the pellet was washed with 0.5 mL of reticulocyte saline (RS) buffer with 25 mM EDTA (4500 rpm, 5 min). A second wash was performed with 0.5 mL of RS buffer (4500 rpm, 5 min). The sample was resuspended in 25 µL of RS buffer and sonicated for 1 min before use as template for real-time PCR. Results obtained by the real-time PCR antibiogram were verified by culture in LB broth and on LB-agar plates with antibiotic discs (Information S1).

### Reticulocyte Saline Buffer

A solution consisting of 130 mM NaCl, 7.4 mM MgCl_2_, and 5 mM KCl was buffered to a pH of 7.35 with 10 mM Na-HEPES.

### Antibiotic 1x Concentrations

The 1x antibiotic concentrations used for the purposes of this investigation were as follows: chloramphenicol: 25 µg/mL in EtOH, kanamycin: 25 µg/mL in DI water, and spectinomycin: 50 µg/mL in water.

### Real-time PCR

Colony PCR was performed with a Bio-Rad iQ5 Real-time PCR Detection System and Stratagene Brilliant SYBR Green qPCR Core Kit. The 16S rRNA gene primers (Invitrogen) used are shown in Table 1 and a 58°C melting temperature was determined optimal for annealing. In accordance with the Stratagene Manual, 2 µL of template was used, taken directly from the prepared protocol described above.

### Sequencing of Amplicons

16S rDNA amplicons were purified with a QUIquick PCR Purification Kit (Qiagen) and sequencing was performed at the UC Berkeley DNA Sequencing Facility using a 48-capillary Applied Biosystems 3730xl DNA Analyzer. The forward primer of the PCR reaction was utilized as the sequencing primer and results were analyzed using the Basic Local Alignment Search Tool (BLAST), provided by the National Center for Biotechnology Information (NCBI). The determined amplicon sequences are reported in the Information S1.

### Simulation of Real-time PCR Antibiogram Threshold

The simulation depicted in Fig. 1 was established using parameters for a typical real-time PCR detection system with decreasing polymerase efficiency, depleting reagent concentrations, and the saturation of fluorescent signal. Simulation parameters were optimized using experimental data.

## Supporting Information

Figure S1 **Protocol optimization for the Bio-Rad iQ5 PCR detection system.** a) A limit of detection (LOD) of 2×10^4^ CFU/mL was determined for colony PCR, although lower limits have been reported. b) Optimization of our experimental protocol necessitates an incubation time of 8+ hours to differentiate bacterial growth from the initial concentrations found in septicemia.
(TIF)

Figure S2 **Additional susceptibility testing with kanamycin resistant E. coli.** a) Additional susceptibility testing was performed for kanamycin resistant E. coli spiked in whole blood. Real-time amplification curves were run in triplicate. b) The corresponding ΔC_t_ values for the real-time amplification curves. ΔC_t_ values >3.0 were assigned to indicate susceptibility, while ΔC_t_ values <3.0 designate resistance. *P<0.05 for multiple comparisons by the Holm *t* Test. t = 0 signifies initial bacterial levels without incubation and negative curves denote sample preparation without bacteria.
(TIF)

Figure S3 **Probability Density Function for statistical comparison of spectinomycin ΔC_t_ values in** **Figure 2D and 2E** **.** A Gaussian ratio distribution was performed between the spectinomycin and negative ΔC_t_ values in both Figures 2D (susceptible) and 2E (spectinomycin resistant). A two-sample Kolmogorov-Smirnov test was performed in MATLAB to compare the distributions. The null hypothesis for this test states that the distributions are from the same, continuous distribution. Our analysis rejects the null hypothesis at a significance level much less than 0.001%. Thus spectinomycin ΔC_t_ values between susceptible and spectinomycin resistant E. coli confirmed a significant statistical difference.
(TIF)

Information S1 **Amplicon sequences used for BLAST determination, verification of the real-time PCR antibiogram results via culture in LB broth and on LB-agar plates with antibiotic discs, and statistical comparison of spectinomycin ΔC_t_ values between susceptible and spectinomycin resistant E. coli in** **Figure 2D and 2E** **.**
(DOC)
