# Supplementary figures and images for: A Real-Time PCR Antibiogram for Drug-Resistant Sepsis
Source: PLoS One. 2011 Dec 2;6(12):e28528. doi: 10.1371/journal.pone.0028528 (PMC3229610; doi:10.1371/journal.pone.0028528)

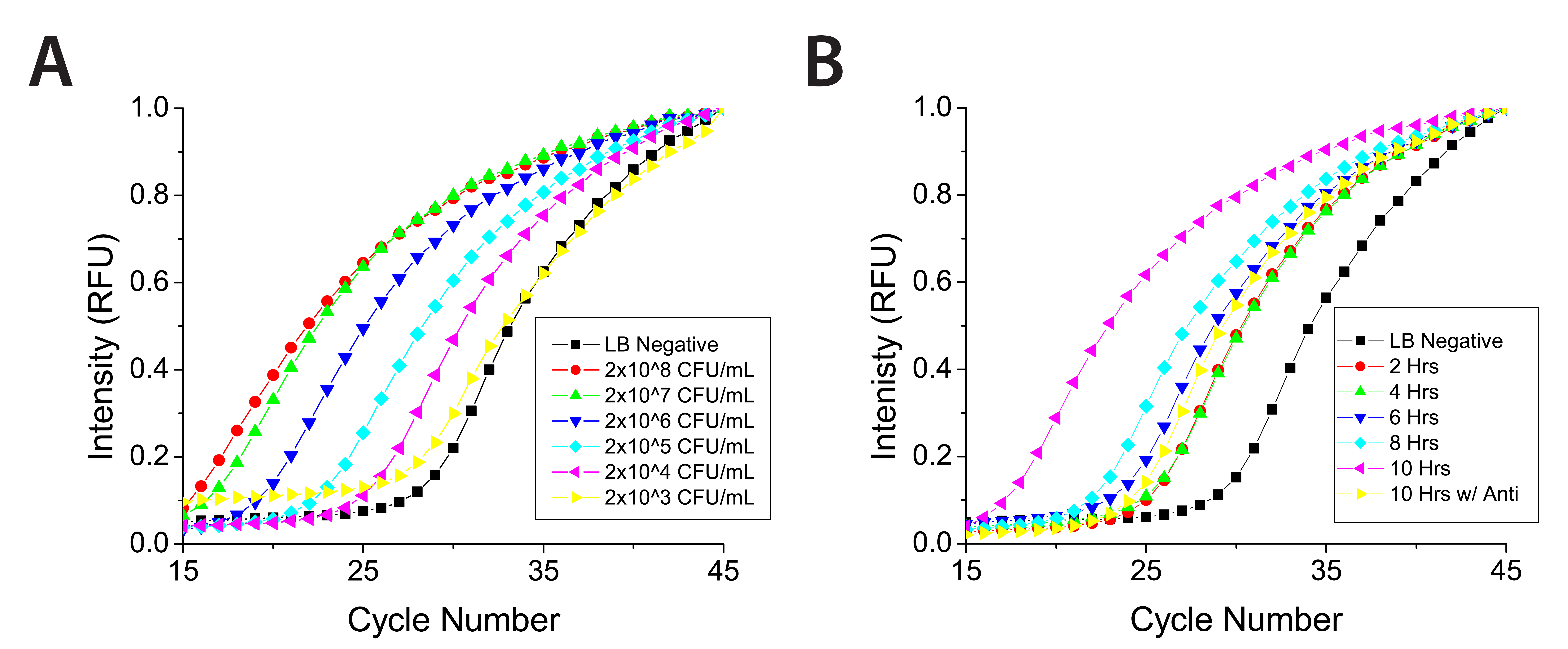

Supplement: Figure S1 — Protocol optimization for the Bio-Rad iQ5 PCR detection system. a) A limit of detection (LOD) of 2×10^4^ CFU/mL was determined for colony PCR, although lower limits have been reported. b) Optimization of our experimental protocol necessitates an incubation time of 8+ hours to differentiate bacterial growth from the initial concentrations found in septicemia. (TIF) [file pone.0028528.s001.tif]

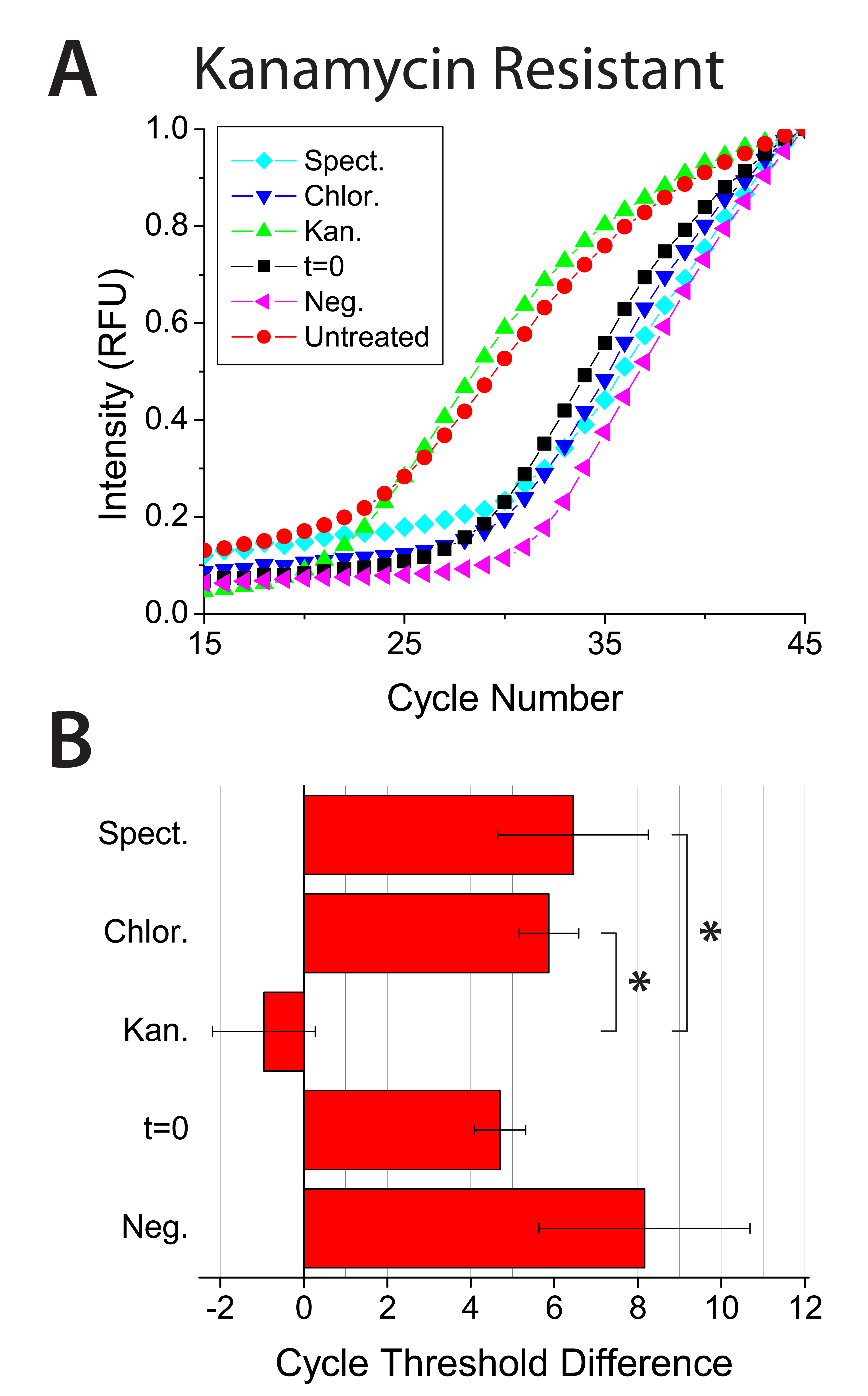

Supplement: Figure S2 — Additional susceptibility testing with kanamycin resistant E. coli. a) Additional susceptibility testing was performed for kanamycin resistant E. coli spiked in whole blood. Real-time amplification curves were run in triplicate. b) The corresponding ΔCt values for the real-time amplification curves. ΔCt values >3.0 were assigned to indicate susceptibility, while ΔCt values <3.0 designate resistance. *P<0.05 for multiple comparisons by the Holm t Test. t = 0 signifies initial bacterial levels without incubation and negative curves denote sample preparation without bacteria. (TIF) [file pone.0028528.s002.tif]

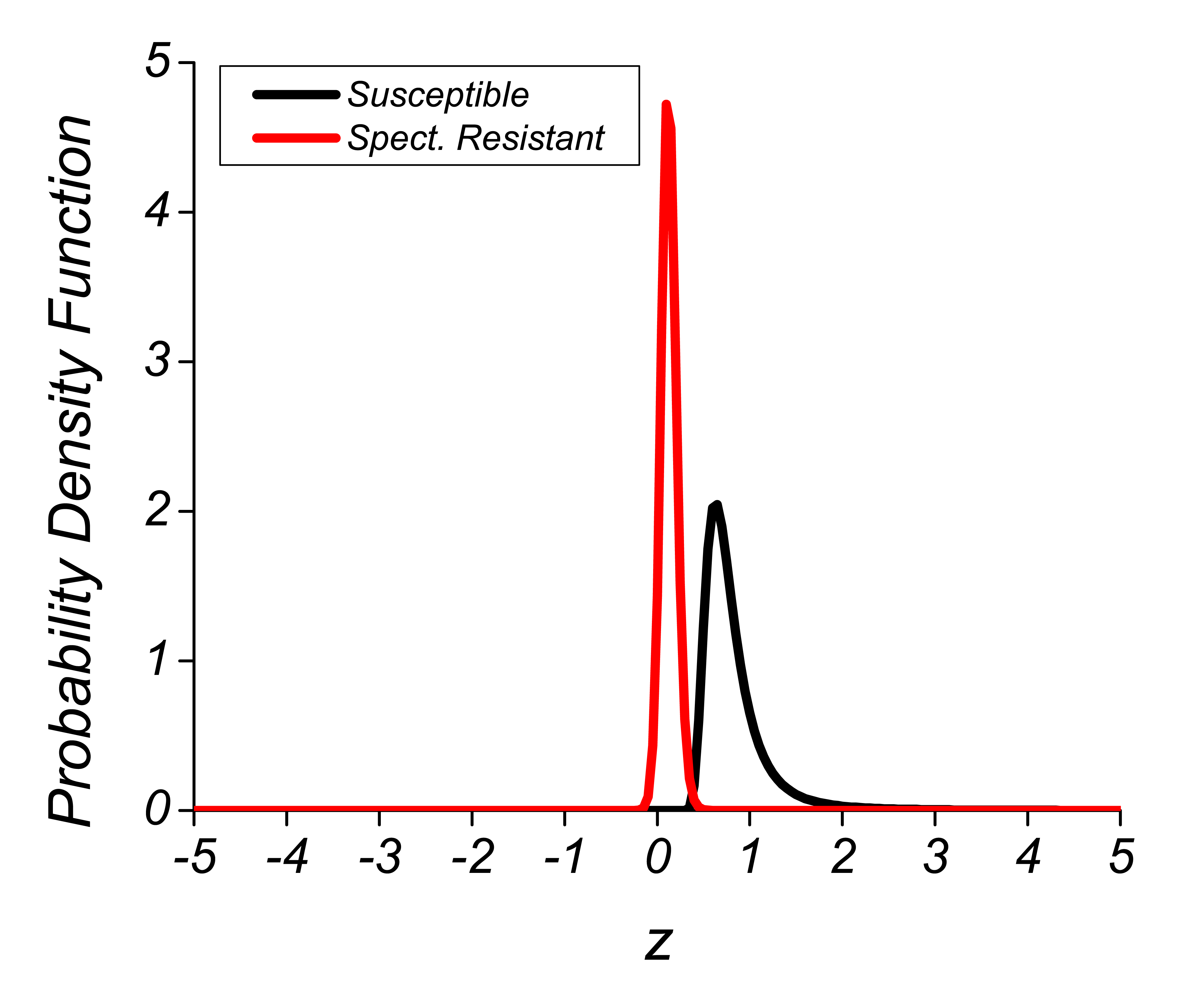

Supplement: Figure S3 — Probability Density Function for statistical comparison of spectinomycin ΔCt values in Figure 2D and 2E . A Gaussian ratio distribution was performed between the spectinomycin and negative ΔCt values in both Figures 2D (susceptible) and 2E (spectinomycin resistant). A two-sample Kolmogorov-Smirnov test was performed in MATLAB to compare the distributions. The null hypothesis for this test states that the distributions are from the same, continuous distribution. Our analysis rejects the null hypothesis at a significance level much less than 0.001%. Thus spectinomycin ΔCt values between susceptible and spectinomycin resistant E. coli confirmed a significant statistical difference. (TIF) [file pone.0028528.s003.tif]
