# Supplementary material for: A Real-Time PCR Antibiogram for Drug-Resistant Sepsis
Source: PLoS One. 2011 Dec 2;6(12):e28528. doi: 10.1371/journal.pone.0028528 (PMC3229610; doi:10.1371/journal.pone.0028528)
Supplement: Information S1 — Amplicon sequences used for BLAST determination, verification of the real-time PCR antibiogram results via culture in LB broth and on LB-agar plates with antibiotic discs, and statistical comparison of spectinomycin ΔCt values between susceptible and spectinomycin resistant E. coli in Figure 2D and 2E . (DOC) [file pone.0028528.s004.doc]

**A Real-time PCR Antibiogram for Drug-resistant Sepsis**

John R. Waldeisen^†^, Tim Wang^†^, Debkishore Mitra, Luke P. Lee*

Department of Bioengineering, University of California, Berkeley, California, United States of America

^†^Authors Contributed Equally

*Corresponding Author

Luke P. Lee

408C Stanley Hall

University of California

Berkeley, CA

Phone: (510) 642-5855

Fax: (510) 642-5835

Email: lplee@berkeley.edu

**SUPPORTING INFORMATION:**

**SUPPORTING DATA:**

*Sequences used for BLAST Determination*

EC: Score: 285 E-Value: 2e-74 (215 Bases)

CTGGNAAACTGCCTGATGGAGGGGGATAACTACTGGAAACGGNAGCTAATACCGCATAACGNCGCAAGANCAAAGAGGGGGACCTTCCGGCCTCTTGCCATCNGATGNGCCCAGANGGGANNAGNTAGNAGGTGGGGTAANGGNTCANCNAGNNNANGATCCCTAGNTGGNCTGAGAGGATGANCAGNCACNNTGNAANTGANANACGGTCCAGA

PF: Score: 226 E-Value: 1e-56 (196 Bases)

ACGGNCGCNAGNTNNCTCTCGTAGGACGTATGCNGNATTAGCGNCCGTTTCCGAANGNTATCCCCCACTACCAGGNAGATTCCTAGGCTTTACTCACCCGTCCGCCGNNCTCAAGAGAAGCAAGCTTCTCTCTACCGCTCGANTNGNNTGTGNNAGGNCTGCCGACAGCGTTCNCTCAGANCCATGANAANAANCT

PV: Score: 129 E-Value 5e-30 (216 Bases)

ATGGGGATCTGCCCGATAGAGGGGGANAACTANTGNAAACGNNAGNTNATACCGAATGACGTCNACGGANCAAAGAAGGNGNTCTNCNGANCTTGNNCTATCNGATGAACNCCTCTGNNANNAGCTAGCAGGTGAGGTAATGGNTCACCNANNNNANGATCTCTANCTGGACTGAGAGGATGATCAGNCNCANTGGNGCTGANACANGGCCCACAG

ML: Score 363 E-Value: 1e-97 (257 Bases)

GGNGAANGGGNGAGTAANANGTGAGTAACCTGNNCTTAACTCTGGGATAAGCCTGGGAAANTGGGTCTAATANCGGATAGGAGNGTCCACCGNATGGNGGGTGTTGGAAAGATTTATCGGNTTTGGATGGACTCGNGGNCTATCAGNTTGTTGGTGAGGTAATGGCTCANCAAGGNGACGACGGGNAGCCGGNCTGAGAGGGTGACCGGNCACACTGGGACTGAGACACGGCCCAGANTCCNANNGNANGNAGNAGA

SL: Score: 105 E-Value: 8e-23 (234 Bases)

GACTAANATGCGGGTAANCNGGNNATCNNAAGGGGNNAACAAAAGGAAAAAGGTGCNAANACCGGATAACAATCGAAACCNCATGGNTTCGTTTNGAAAGGNGCTTTACNGGGNCNCCGATGNATGGANNCGNGGTGCNTNAGATAGTNGGTGAGGTAANGGCTCACCAAGGNCACNANGNATANNNGANCTGAGAGGNNGATCGGGCANATTGGGACTGACANACGGNCNAAA

| **Table S1: Verification of real-time PCR antibiogram** | | | | | | | | |
| --- | --- | --- | --- | --- | --- | --- | --- | --- |
|  |  |  | Time to Visual (Hours) | | | | | |
|  |  |  | 0 | 12 | 24 | 36 | 48 | 60 |
| Susceptible E. coli | LB Broth | Spect. | - | - | - | - | - | - |
|  |  | Chlor. | - | - | - | - | - | - |
|  |  | Kan. | - | - | - | - | - | - |
|  |  | Untreat | - | - | - | X | X | X |
|  |  | Neg. | - | - | - | - | - | - |
|  | Antibiotic Discs | Spect. | - | - | - | - | - | - |
|  |  | Chlor. | - | - | - | - | - | - |
|  |  | Kan. | - | - | - | - | - | - |
|  |  | Untreat | - | - | - | - | - | X |
|  |  | Neg. | - | - | - | - | - | - |
| Spectinomycin Resistant | LB Broth | Spect. | - | - | - | X | X | X |
|  |  | Chlor. | - | - | - | - | - | - |
|  |  | Kan. | - | - | - | - | - | - |
|  |  | Untreat | - | - | - | X | X | X |
|  |  | Neg. | - | - | - | - | - | - |
|  | Antibiotic Discs | Spect. | - | - | - | - | - | X |
|  |  | Chlor. | - | - | - | - | - | - |
|  |  | Kan. | - | - | - | - | - | - |
|  |  | Untreat | - | - | - | - | - | X |
|  |  | Neg. | - | - | - | - | - | - |
| Kanamycin Resistant | LB Broth | Spect. | - | - | - | - | - | - |
|  |  | Chlor. | - | - | - | - | - | - |
|  |  | Kan. | - | - | - | X | X | X |
|  |  | Untreat | - | - | - | X | X | X |
|  |  | Neg. | - | - | - | - | - | - |
|  | Antibiotic Discs | Spect. | - | - | - | - | - | - |
|  |  | Chlor. | - | - | - | - | - | - |
|  |  | Kan. | - | - | - | - | - | X |
|  |  | Untreat | - | - | - | - | - | X |
|  |  | Neg. | - | - | - | - | - | - |
| MIC Determination | Microdilution in LB Broth | 10x Spect. | - | - | - | - | - | - |
|  |  | 1x Spect. | - | - | - | - | - | - |
|  |  | 1/10x Spect. | - | - | - | - | - | X |
|  |  | 1/100x Spect. | - | - | - | X | X | X |
|  |  | Untreat | - | - | - | X | X | X |
|  |  | Neg. | - | - | - | - | - | - |
|  | Antibiotic Discs | 10x Spect. | - | - | - | - | - | - |
|  |  | 1x Spect. | - | - | - | - | - | - |
|  |  | 1/10x Spect. | - | - | - | - | - | - |
|  |  | 1/100x Spect. | - | - | - | - | - | X |
|  |  | Untreat | - | - | - | - | - | X |
|  |  | Neg. | - | - | - | - | - | - |

*Comparison of spectinomycin ΔC_t_ values between susceptible and spectinomycin resistant E. coli in Figure 2D and 2E:*

A Gaussian ratio distribution was performed between the spectinomycin and negative ΔC_t_ values in both Figures 2D (susceptible) and 2E (spectinomycin resistant). The distributions are assumed to be normal and not correlated. Since the ΔC_t_ values both have a non-zero mean, the Hinkley distribution was utilized where the probability density function of the ratio between two normal variables (Z=X/Y) is given by [1]:

$$P(z)=\frac{b(z)\cdot c(z)}{a^{3}(z)}\frac{1}{\sigma_{X}\sigma_{Y}\sqrt{2\pi}}\left[2\Phi\left(\frac{b(z)}{a(z)}\right)-1\right]+\frac{1}{\pi\sigma_{X}\sigma_{Y}a^{2}(z)}e^{-\frac{1}{2}\left(\frac{\mu_{X}^{2}}{\sigma^{2}_{X}}+\frac{\mu_{Y}^{2}}{\sigma^{2}_{Y}}\right)}$$

where $a(z)=\sqrt{\frac{1}{\sigma^{2}_{X}}z^{2}+\frac{1}{\sigma^{2}_{Y}}}$

$$b(z)=\frac{\mu_{X}}{\sigma^{2}_{X}}z+\frac{\mu_{Y}}{\sigma^{2}_{Y}}$$

$$c(z)=e^{\frac{1}{2}\frac{b^{2}(z)}{a^{2}(z)}-\frac{1}{2}\left(\frac{\mu^{2}_{X}}{\sigma^{2}_{X}}+\frac{\mu^{2}_{Y}}{\sigma^{2}_{Y}}\right)}$$

$$\Phi(z)=\int_{-\infty}^{z} \frac{1}{\sqrt{2\pi}}e^{-\frac{1}{2}u^{2}}du=\frac{1}{2}\text{erfc}\left(-\frac{z}{\sqrt{2}}\right)$$

Figure S3 depicts the probability density functions for the comparison of Figures 2D and 2E. A two-sample Kolmogorov-Smirnov test was performed in MATLAB to compare the distributions. The null hypothesis for this test states that the distributions are from the same, continuous distribution. Our analysis rejects the null hypothesis at a significance level much less than 0.001%. Thus spectinomycin ΔC_t_ values between susceptible and spectinomycin resistant E. coli confirmed a significant statistical difference.

**References:**

[1] Hinkley DV, (1969) On the ratio of two correlated normal random variables. Biometrika 56: 635-639.
